# Arterial Stiffness and Left Ventricular Diastolic Function in Subjects with Euthyroidism

**DOI:** 10.31083/j.rcm2305150

**Published:** 2022-04-26

**Authors:** Lijuan Yang, Xiuqin Sun, Hong Tao, Yi Zhao

**Affiliations:** ^1^Department of Endocrinology and Metabolism, Beijing Anzhen Hospital, Capital Medical University, 100029 Beijing, China

**Keywords:** thyroid-stimulating hormone, brachial-ankle pulse wave velocity, arterial stiffness, left ventricular diastolic function, thyroperoxidase antibody

## Abstract

**Objective:**

Previous literature has suggested that the cardiovascular risk factors associated with subclinical hypothyroidism (SCH) may be found in subjects with euthyroidism, but research relating to increased arterial stiffness (AS) and left ventricular (LV) diastolic dysfunction, which have been proven to exist in patients with SCH, is limited in patients with euthyroidism. The aim of this study was to investigate this.

**Methods:**

A total of 249 participants with euthyroidism were divided into two groups based on their thyroid-stimulating hormone (TSH) levels: Group A (TSH level ranging from 0.49 to 2.5 mIU/L, n = 170) and Group B (TSH level ranging from 2.5 to 4.91 mIU/L, n = 79). The Cardiovascular Profiling System through brachial-ankle pulse wave velocity (baPWV) was used to assess AS, and the LV function was evaluated using Color-Doppler-Echocardiography. The Student’s unpaired *t*-test and Pearson’s χ^2^ test were conducted to compare the clinical parameters. Spearman’s correlation analysis and multiple logistic regression analysis were used to analyze the association between thyroid function, baPWV, and LV diastolic function parameters.

**Results:**

Significant differences existed between the two groups in free triiodothyronine (${\mathrm{fT}}_{3}$) values and systolic blood pressure (BP) (*p <* 0.05). When compared with Group A, the baPWV was higher, the A wave increased, and the E/A ratio was lower in Group B (*p <* 0.01). The multiple logistic regression analysis showed that ${\mathrm{fT}}_{3}$ was associated with a higher baPWV (*p <* 0.001). The E/A ratio was directly correlated with TSH, ${\mathrm{fT}}_{3}$, and baPWV (*p *< 0.05), and diastolic BP was significantly directly correlated with the E/A ratio (*p <* 0.05). Thyroperoxidase antibody was not a significant variable in the regression analysis (*p >* 0.05).

**Conclusions:**

An association was found between thyroid function, baPWV, and the E/A ratio in subjects with euthyroidism. Further study is needed to confirm these conclusions.

## 1. Introduction

Thyroid dysfunction presents as one of the most frequently occurring endocrine diseases [1]. It has been reported that the prevalence of subclinical hypothyroidism (SCH) ranges from 0.4%–20% [2, 3]. Previous studies have suggested that SCH may worsen many risk factors for cardiovascular disease (CVD), including hypertension, atherosclerosis, dyslipidemia, and abnormal endothelial function, and even result in CVD directly, such as pericarditis [4, 5].

Arterial stiffness (AS) appears to be an important risk factor for atherosclerosis and hypertension, as well as in the process of left ventricular (LV) diastolic dysfunction (DD) [6, 7]. A growing number of studies have indicated that patients with SCH exhibit increased AS and impaired LV diastolic function when compared with people with euthyroidism [8, 9].

As noted by the National Health and Nutrition Examination Survey III from the United States of America (USA), the thyroid-stimulating hormone (TSH) upper reference limits may be skewed by thyroperoxidase antibody (TPO-Ab)-negative individuals with occult autoimmune thyroid dysfunction [10]. This may signify that some people with mild hypothyroidism, including SCH, were not identified and were treated as if they had normal thyroid function. Therefore, people with TSH levels close to the upper limit may suffer from similar adverse effects as people with SCH.

The literature revealed that decreased endothelium-dependent vasodilatation (a sign of endothelial dysfunction), increased blood pressure (BP), and dyslipidemia were observed in people with high–normal TSH levels [11, 12, 13]. However, AS and LV DD in patients with high–normal TSH levels have not yet been adequately studied. Pulse wave velocity (PWV) is an index measuring AS by measuring the speed of a pulse wave along an artery. A higher PWV indicates increased stiffness in the arterial wall. Lambrinoudaki *et al*. [14] reported that a high–normal TSH level is associated with increased PWV, but the study subjects were all healthy postmenopausal women. Sandra *et al*. [15] suggested that cardiac diastolic abnormalities occur in patients with autoimmune thyroiditis when their serum TSH levels are still within the normal range, but the sample size was small (n = 45).

Subjects with high–normal TSH levels frequently progress to hypothyroidism later in life [16], and an evaluation of AS in this group was warranted. As a result, the present study was conducted to confirm the hypothesis that AS might be greater in people with high–normal TSH levels when compared with people with normal TSH levels, using a simple measurement, i.e., brachial-ankle PWV (baPWV) in a larger sample size. Indices indicating the LV diastolic function were analyzed.

## 2. Materials and Methods

### 2.1 Study Participants

This retrospective study was conducted to examine the AS and the LV diastolic function in subjects with euthyroidism. The participants were collected by searching the computerized database of patients hospitalized in the Department of Endocrinology and Metabolism at our hospital from December 2015 to November 2021. A total of 425 participants who underwent a measurement for AS were included for eligibility assessment. The district in which the study was conducted is not an iodine-deficient district.

The following exclusion criteria were used: (1) incomplete personal medical and drug treatment history (missing vital data); (2) thyroid function not within the normal range or a history of thyroid disease or therapy; (3) the use of drugs known to affect thyroid function, e.g., metformin [17], glucocorticoids, dopamine agonists, rexinoids, carbamazepine, and metyrapone [18]; (4) a previous history of serious cardiovascular events, e.g., ischemic heart disease or heart failure, stroke, or chronic obstructive peripheral arteriopathy (ankle–brachial index <0.9 or detected by Doppler ultrasonography); (5) other serious chronic diseases, a recent history of acute illness, malignant disease, or severely impaired renal function, i.e., estimated glomerular filtration rate (eGFR) <30 mL/min/1.73 $m^{2}$; (6) drug or alcohol abuse.

A total of 249 subjects with euthyroidism were included in the final analysis. To evaluate the AS and LV functions in relation to the TSH level, all patients were grouped according to their serum TSH level into the normal TSH group (Group A, 0.49 mIU/L ≤ TSH ≤ 2.5 mIU/L) and the high–normal TSH group (Group B, 2.5 mIU/L < TSH ≤ 4.91 mIU/L). The cutoff point was set according to studies researching subjects with euthyroidism [14, 17].

The study was conducted in accordance with the declaration of Helsinki and approved by our hospital ethical committee.

### 2.2 Clinical Measurements and Biochemical Analysis

The anthropometric indices (body weight and height) were measured by professional investigators using a standard protocol. Height was assessed to the nearest 1 cm and body weight with light clothing to the nearest 0.1 kg. The body mass index (BMI) was calculated as the body weight (kg) divided by the square of the body height (m). Office BP was measured on the right arm using an automatic BP monitor (J30 [±3 mmHg], Omron Healthcare Co., Ltd., Kyoto, Japan) after a minimum of 5 min of rest in a sitting position. The sociodemographic characteristics, including age, gender, smoking status, individual history of disease (CVD, stroke, hypertension, dyslipidemia, type 2 diabetes, and thyroid disorders), and detailed drug treatment were collected from participants’ medical records.

The venous blood samples were collected between 6 AM and 8 AM after fasting for at least 10 h for the measurement of thyroid function, serum lipid profile, and renal function parameters. Serum concentrations of TSH (normal range: 0.49–4.91 mIU/L), free triiodothyronine (${\mathrm{fT}}_{3}$) (normal range: 3.28–6.47 pmol/L), free thyroxine (${\mathrm{fT}}_{4}$) (normal range: 7.64–16.03 pmol/L), and TPO-Ab (normal range: 0–9 IU/mL) were measured using an automatic analyzer and the chemiluminescence method (UniCel Dxl 800 Access Immunoassay System, Beckman Coulter, FUL, CA, USA) using reagents from Beckman Coulter. The serum total cholesterol (TC), high-density lipoprotein cholesterol (HDL-C), low-density lipoprotein cholesterol, triglyceride, and uric acid (UA) were measured using colorimetry methods; creatinine (Cr) was measured using the picric acid method; and fasting blood glucose was measured using the hexose kinase method. These measurements were performed on an automated instrument (cobas® 8000; Roche Diagnostics GmbH, Mannheim, Germany) using reagents from Roche Diagnostics. The intra- and inter-assay coefficients of variation were below 5% for the above parameters.

### 2.3 Arterial Wall Stiffness and Left Ventricular Function Assessment

The Cardiovascular Profiling System Colin BP-203RPE III (Omron Healthcare Co., Ltd., Dalian, China) operated by well-trained observers was used to assess AS noninvasively after rest for at least 10 min in a lying position in the Endocrinology and Metabolism Department ward. Indices reflecting AS were evaluated through the baPWV (normal range <14 m/s), which expresses the speed of a pulse wave traveling between the brachial and the ankle arteries and increases with increased stiffness of the arterial wall.

The LV function was evaluated using color Doppler echocardiography (Epiq 7c, Philips Healthcare, Bothell, WA, USA) at rest by professional medical workers. The parameters involved were as follows: (1) interventricular septum thickness (IVST, cm) and the LV posterior wall thickness (LVPWT, cm); (2) the LV end-diastolic diameter (LVEDD, cm) and the LV end-systolic diameter (cm); (3) the left atrial diameter (cm); (4) the peak early diastolic phase (cm/s) and the late diastolic phase (cm/s) mitral inflow velocities; and (5) the LV ejection fraction (%).

The LV geometry was assessed with the LV mass index (LVMI) (g/$m^{2}$) (over 115 g/$m^{2}$ in males and over 95 g/$m^{2}$ in females was considered as LV hypertrophy) and the relative wall thickness (RWT) using the method described by Yoshida *et al*. [19]. The LV mass (normal range: male: 77.6–194.0 g; female: 57.1–157.5 g) was calculated from the end-diastolic IVST, LVPWT, and LVEDD using the following equation:

LV (g) = 0.80 × 1.04 × (${\mathrm{[IVST + LVPWT + LVEDD]}}^{3}$ ${\mathrm{– [LVEDD]}}^{3}$) + 0.6

The LV mass was corrected for the body surface area (BSA) to obtain LVMI. The BSA was calculated from height (cm) and weight (kg) using the following equation from DuBois & DuBois [20]:

BSA ($m^{2}$) = 0.007184 × ${\mathrm{weight}}^{0.425}$ × ${\mathrm{height}}^{0.725}$

The RWT was calculated from LVPWT and LVEDD using the following equation [19]:

RWT = (2 × LVPWT) ÷ LVEDD

### 2.4 Statistical Analysis 

The distribution of continuous variables was evaluated using the Shapiro–Wilk test. Data with a normal distribution or approximate normal distribution were expressed as mean and standard deviation, and other data were expressed as median and interquartile range. Categorical variables were presented as absolute numbers with percentages.

For comparing clinical characteristics, the baPWV, and the parameters of the LV function between the two groups, the Student’s unpaired *t*-test was performed for continuous variables and Pearson’s χ^2^ test was used for categorical variables. For data with a non-normal distribution, a nonparametric test was conducted. Spearman’s correlation coefficient was calculated to evaluate the crude relationships between thyroid function index, baPWV, and the significantly different LV function parameters. Multiple logistic regression analysis was conducted to analyze the association between the thyroid function index, baPWV, and the significantly different LV function parameters.

We used two multivariate regression models. The first model included gender; age; BMI; resting heart rate; BP; antihypertensive, anti-diabetes, or statin treatment; and smoking status. The second model included variables that achieved statistical significance in the first model and the other remaining variables. Due to collinearity, Cr was not included in the model with eGFR. A dummy variable was set for transforming the categorical variable smoking status, and never having smoked was identified as the control. The results of the regression analysis were represented as odds ratios with 95% confidence intervals.

A *p* value < 0.05 was considered statistically significant. The Statistical Package for Social Sciences version 23.0 (IBM, Armonk, NY, USA) was used for calculations and statistical analyses.

## 3. Results

### 3.1 Participants’ Characteristics

The clinical characteristics of the two groups are shown in Table 1. The gender, age, BMI, resting heart rate, diastolic BP (DBP), serum lipid profile, blood glucose, Cr, and UA were comparable between the groups (*p >* 0.05). No significant differences were observed regarding the proportion of patients smoking or receiving antihypertensive, anti-diabetes, or lipid-lowering therapy (*p >* 0.05). Significant differences were found between the two groups in terms of the systolic BP (SBP) (*p <* 0.05), eGFR (*p <* 0.05), and ${\mathrm{fT}}_{3}$ values (*p <* 0.001), whereas no significant differences were found in the ${\mathrm{fT}}_{4}$ level and positive TPO-Ab (*p >* 0.05).

**Table 1.** **Study groups’ characteristics**.

		Normal TSH group (group A) (n = 170)	High-normal TSH group (group B) (n = 79)	*p* value
Sex, male, n (%)		105 (61.8%)	47 (59.5%)	0.732
Age, years		48.9 ± 11.7	51.8 ± 13.8	0.086
BMI, Kg/$m^{2}$		27.6 ± 4.6	27.5 ± 4.7	0.926
Resting heart rate, beats/min		74.4 ± 13.1	75.5 ± 12.2	0.514
Office SBP, mmHg		137.2 ± 16.2	141.8 ± 17.4	0.046
Office DBP, mmHg		83.9 ± 11.8	85.0 ± 13.4	0.505
Antihypertensive treatment, n (%)		151 (88.8%)	67 (84.8%)	0.372
Anti-diabetes treatment, n (%)		90 (52.9%)	48 (60.8%)	0.248
Statin treatment, n (%)		95 (55.9%)	42 (53.2%)	0.688
Smoking, n (%)				0.334
	Never	96 (56.5%)	50 (63.3%)	
	Previous	11 (6.5%)	7 (8.9%)	
	Current	63 (37.1%)	22 (27.8%)	
TC, mmol/L		5.1 ± 1.1	5.1 ± 1.1	0.590
HDL-C, mmol/L		1.2 ± 0.3	1.1 ± 0.3	0.386
LDL-C, mmol/L		3.0 ± 0.9	2.9 ± 0.9	0.546
TGs, mmol/L		2.0 ± 1.2	2.1 ± 1.2	0.537
Cr, umol/L		80.6 ± 25.7	81.3 ± 19.3	0.838
eGFR, mL/min/1.73 $m^{2}$		93.1 ± 20.7	87.9 ± 15.8	0.031
UA, umol/L		371.0 ± 107.9	367.2 ± 114.4	0.797
FBG, mmol/L		6.0 ± 1.5	6.1 ± 1.7	0.632
TSH (mIU/L)		1.5 ± 0.5	3.4 ± 0.7	<0.001
fT_3_ (pmol/L)		5.2 ± 0.7	4.3 ± 0.8	<0.001
fT_4_ (pmol/L)		11.4 ± 1.7	11.1 ± 1.9	0.207
TPOAb (+), n (%)		16 (9.4%)	12 (15.2%)	0.179

Data with normal distribution or approximate normal distribution were expressed as mean ± standard deviation (S.D.). Categorical variables were recorded as absolute numbers with percentages. BMI, body mass index; SBP, systolic blood pressure; DBP, diastolic blood pressure; TC, total cholesterol; HDL-C, high-density lipoprotein cholesterol; LDL-C, low-density lipoprotein cholesterol; TGs, triglycerides; Cr, creatinine; eGFR, estimated glomerular filtration rate; UA, uric acid; FBG, fasting blood glucose; TSH, thyroid-stimulating hormone; fT_3_, free triiodothyronine; fT_4_, free thyroxine; TPOAb, thyroperoxidases antibody. TPOAb (+) was defined as serum TPOAb higher than their upper limit of reference ranges.

The data relating to the baPWV and LV functions are illustrated in Table 2. When compared with the normal group, the baPWV and A wave were significantly higher and the E/A ratio was lower in the high–normal group (*p <* 0.01). No significant differences were found in the other LV function indices between the two groups (*p >* 0.05).

**Table 2.** **Comparison of artery stiffness and left ventricular function in study groups**.

		Normal TSH group (group A) (n = 170)	High-normal TSH group (group B) (n = 79)	*p* value
baPWV (m/s)				
	left	16.3 ± 3.6	18.7 ± 5.2	<0.001
	right	16.0 ± 3.3	18.0 ± 4.7	0.001
IVST (mm)		10.3 ± 1.7	10.5 ± 1.8	0.319
LVPWT (mm)		9.6 ± 1.5	9.5 ± 1.5	0.823
LVEDD (mm)		46.4 ± 4.3	46.7 ± 4.7	0.616
LVESD (mm)		29.2 ± 3.4	29.9 ± 4.2	0.156
LAD (mm)		36.2 ± 4.3	36.2 ± 4.7	0.970
E (cm/s)		75.1 ± 16.5	72.7 ± 18.7	0.326
A (cm/s)		74.3 ± 22.5	85.7 ± 18.9	<0.001
E/A		1.1 ± 0.5	0.9 ± 0.3	0.001
LVEF (%)		65.4 ± 3.8	64.3 ± 5.4	0.090
LVMI (g/$m^{2}$)		85.7 ± 20.9	88.7 ± 23.8	0.319
RWT		0.4 ± 0.1	0.4 ± 0.1	0.636

Continuous data with normal distribution or approximate normal distribution were reported as mean ± standard deviation (S.D.). baPWV, brachial-ankle pulse wave velocity; IVST, interventricular septum thickness; LVPWT, left ventricular posterior wall thickness; LVEDD, left ventricular end-diastolic diameter; LVESD, left ventricular end-systolic diameter; LAD, left atrial diameter; E and A, peak early diastolic phase and late diastolic phase mitral inflow velocities; LVEF, left ventricular ejection fraction; LVMI, left ventricular mass index; RWT, relative wall thickness.

### 3.2 Association Between Thyroid Function, Arterial Stiffness, and Left Ventricular Function Parameters

The Spearman’s correlation analysis showed baPWV was negatively correlated with ${\mathrm{fT}}_{3}$ (r = –0.585, *p <* 0.001), but no significant correlation was found with TSH (r = 0.101, *p *= 0.111) or ${\mathrm{fT}}_{4}$ (r = 0.045, *p *= 0.477). The correlation was significant between E/A with baPWV (r = 0.555, *p <* 0.001), TSH (r = 0.235, *p <* 0.001), and fT_3_ (r = –0.554, *p <* 0.001), but not with fT_4_ (r = 0.030, *p *= 0.634), where E/A ratio >1 was represented as 0 and E/A ratio <1 was represented as 1.

The results of the association between the thyroid function and the baPWV assessed by multiple logistic regression analysis are represented in Table 3. The association between the ${\mathrm{fT}}_{3}$ and baPWV values was significant after adjustment for the variables in models 1 and 2 (*p <* 0.001), but no significant association was found between the TSH and baPWV values after adjustment (*p >* 0.05). Additionally, the DBP and SBP were not significantly associated with the baPWV in the analysis, and TPO-Ab and the eGFR were not significant variables in the models (*p >* 0.05). Significant association between age and the baPWV was found (*EXP(B) *= 1.043,* p *= 0.034), but the association between smoking and the baPWV did not reach significance (*EXP(B) *= 1.414,* p *= 0.079).

**Table 3.** **Multiple logistic regression analysis with baPWV as dependent variable (n = 249)**.

			*B*	*EXP(B)*	95% CI of *EXP(B)*		*p* value
TSH							
	Model 1						
		baPWV (left)	–0.103	0.902	0.596	1.364	0.624
		baPWV (right)	–0.329	0.720	0.450	1.152	0.170
	Model 2						
		baPWV (left)	–0.171	0.843	0.567	1.252	0.397
		baPWV (right)	–0.307	0.736	0.474	1.143	0.173
fT_3_							
	Model 1						
		baPWV (left)	–1.986	0.137	0.065	0.288	<0.001
		baPWV (right)	–1.927	0.146	0.066	0.323	<0.001
	Model 2						
		baPWV (left)	–1.900	0.150	0.074	0.303	<0.001
		baPWV (right)	–1.888	0.151	0.071	0.322	<0.001
fT_4_							
	Model 1						
		baPWV (left)	–0.069	0.933	0.731	1.192	0.580
		baPWV (right)	0.067	1.069	0.820	1.395	0.621
	Model 2						
		baPWV (left)	–0.031	0.969	0.769	1.222	0.791
		baPWV (right)	0.118	1.125	0.880	1.440	0.348

BaPWV <18 m/s, represented as 0, baPWV >18 m/s, represented as 1. TSH, thyroid-stimulating hormone; fT_3_, free triiodothyronine; fT_4_, free thyroxine; baPWV, brachial-ankle pulse wave velocity. Multivariable model 1 was adjusted for sex, age, BMI, resting heart rate, BP, antihypertensive/anti-diabetes/statin treatment and smoking situation; Multivariable model 2 was further adjusted for variables reaching statistical significance in the first model and all the rest variables.

The multiple logistic regression analysis was conducted to further assess the association between the baPWV and the E/A ratio, excluding the thyroid function parameters (see Table 4). The results showed a significant association between the baPWV and the E/A ratio in both models (*p <* 0.01) and an association between the DBP and the E/A ratio in model 2 (*p <* 0.05). We then conducted the regression analysis again, including the thyroid function parameters (see Table 5). The analysis showed no significant association between the baPWV and the E/A ratio (*p >* 0.05) and a significant association between ${\mathrm{fT}}_{3}$, TSH, and the E/A ratio, and an association between the DBP and the E/A ratio (*p <* 0.05). Also, TPO-Ab was not found to be a significant variable in the analysis (*p >* 0.05). The association between age and the E/A ratio was significant (*EXP(B) *= 1.085,* p <* 0.001). No significant association was found between gender and the E/A ratio (*EXP(B) *= 0.573, *p *= 0.206, where male was represented as 1 and female was represented as 0.

**Table 4.** **Multiple logistic regression analysis with E/A ratio as dependent variable (n = 249)**.

		*B*	*EXP(B)*	95% CI of *EXP(B)*		*p* value
baPWV (average)						
	Model 1	0.213	1.237	1.079	1.419	0.002
	Model 2	0.240	1.272	1.108	1.459	0.001
DBP						
	Model 1	0.049	1.050	0.997	1.107	0.067
	Model 2	0.035	1.035	1.003	1.069	0.031

E/A ratio >1 represented as 0, E/A ratio <1 represented as 1. The average level of left and right baPWV was calculated and included in the regression analysis. Thyroid function parameters were not included in the regression analysis. baPWV, brachial-ankle pulse wave velocity; DBP, diastolic blood pressure. Multivariable model 1 was adjusted for sex, age, BMI, resting heart rate, BP, antihypertensive/anti-diabetes/statin treatment and smoking situation. Multivariable model 2 was further adjusted for variables reaching statistical significance in the first model and all the rest variables.

**Table 5.** **Multiple logistic regression analysis with E/A ratio as dependent variable (n = 249)**.

		*B*	*EXP(B)*	95% CI of *EXP(B)*		*p* value
TSH						
	Model 1	0.397	1.487	1.028	2.151	0.035
	Model 2	0.424	1.527	1.056	2.209	0.024
fT_3_						
	Model 1	–1.400	0.247	0.126	0.483	<0.001
	Model 2	–1.266	0.282	0.150	0.529	<0.001
fT_4_						
	Model 1	0.090	1.094	0.887	1.350	0.401
	Model 2	0.030	1.031	0.838	1.268	0.774
baPWV (average)						
	Model 1	0.049	1.050	0.904	1.221	0.522
	Model 2	0.088	1.091	0.940	1.267	0.250
DBP						
	Model 1	0.059	1.061	1.003	1.122	0.038
	Model 2	0.045	1.046	1.010	1.083	0.012

E/A ratio >1 represented as 0, E/A ratio <1 represented as 1. Thyroid function parameters were included in the regression analysis. The average level of left and right baPWV was calculated and included in the regression analysis. TSH, thyroid-stimulating hormone; fT_3_, free triiodothyronine; fT_4_, free thyroxine; baPWV, brachial-ankle pulse wave velocity; DBP, diastolic blood pressure. Multivariable model 1 was adjusted for sex, age, BMI, resting heart rate, BP, antihypertensive/anti-diabetes/statin treatment and smoking situation; Multivariable model 2 was further adjusted for variables reaching statistical significance in the first model and all the rest variables.

## 4. Discussion

The main finding of the current study is that, when compared with the normal group, the baPWV was higher and the E/A ratio was lower in the high–normal group. We also provided evidence of the association between the thyroid function, increased AS, and LV DD in subjects with euthyroidism after adjustment for confounders.

The PWV is a critical index for evaluating AS and is widely used for the noninvasive assessment of early atherosclerosis [8], and baPWV could reflect the comprehensive PWV of the thoracic aorta, abdominal aorta, and part of the lower extremity arteries. In recent years, the concept of vascular failure was put forward by Japanese researchers [21]. Regarded as a new physiological diagnostic criterion for vascular failure, the optimal cutoff value of baPWV for predicting CVD was suggested to be 18 m/s [22].

A possible explanation for the elevated baPWV in the high–normal group follows. First, it is well known that dyslipidemia is an important factor leading to atherosclerosis and lipid homeostasis is influenced by thyroid hormones [23]. Previous studies have indicated correlations between TSH and dyslipidemia in subjects with euthyroidism [12]. Second, Lekakis revealed that flow-mediated vasodilatation is impaired in patients with high–normal serum TSH levels, which would influence the elastic behavior of the arteries and result in increased AS [11, 24]. Third, as noted in the National Health and Nutrition Examination Survey III [10], the prevalence of TPO-Ab increases with an increase in the TSH level. However, even when TSH was >10 mIU/L, no thyroid autoantibodies were detected in 31% of men and 11% of women. This means when acquiring a TSH reference range through a large sample survey of the population, if thyroid dysfunction was only excluded by the prevalence of thyroid autoantibodies, this would result in subjects with thyroid dysfunction and negative thyroid autoantibodies being regarded as normal. Therefore, the TSH upper reference limits may be skewed, and subjects with high–normal TSH levels may exhibit a similar presentation to patients with SCH. Therefore, it appeared reasonable that the baPWV would be higher in subjects with high–normal serum TSH levels.

The present research proceeded to study the indices relating to the LV diastolic function. The PWV and AS increase under the influence of aging, hypertension, and atherosclerosis, which leads to an increase in the BP, pulse pressure, and LV load with hypertrophy and LV DD [25, 26]. An increase in the A wave and a decrease in E/A ratio might reflect early LV DD [27]. Studies on the diastolic function in patients with SCH have showed an increased A wave, reduced E/A ratio, and prolonged isovolumic relaxation time when compared with healthy subjects [28, 29]. The current study showed an increased A wave and a decreased E/A ratio in the high–normal TSH group, which is supported by the underlying mechanism and is in accordance with the study in patients with SCH.

The effect of the thyroid gland on the cardiovascular system is primarily exerted through biologically active $T_{3}$. The related mechanism includes two aspects: (1) the genomic effect (increased gene expression of sarcoplasmic reticulum calcium ATPase [SERCA] or cardiac α-myosin heavy chain and upregulation of the potassium channel through the thyroid hormone receptor-α); and (2) the non-genomic effect (increased nitric oxide [NO] synthesis and release from the endothelial cells) [30, 31, 32]. Previous studies found that reduced calcium reuptake into the sarcoplasmic reticulum from decreased expression of SERCA is involved in LV DD in SCH [30]. A reduction in NO was found to be associated with increased AS in SCH [32]. The activity of type 3 deiodinase was stimulated up to five-fold in hypertrophic ventricular tissue, which converts $T_{4}$ and $T_{3}$ to the inactive compounds ${\mathrm{reverse-T}}_{3}$ and 3, 3’-$T_{2}$, respectively [33].

In the present study, ${\mathrm{fT}}_{3}$ was lower in the high–normal group, and the association analysis after adjustment for confounders also proves the pivotal role of $T_{3}$ in cardiovascular system dysfunction. We could also infer from our investigation that, by affecting BP, increased AS could result in LV DD. In addition, the E/A ratio was found to be correlated with TSH levels, which is consistent with the findings of Sandra [15]. We speculated that some indices, such as the E/A ratio, may also be affected by the local generation of $T_{3}$ from circulating $T_{4}$. As TSH levels were more sensitive than $T_{4}$, the association to $T_{4}$ may be explained. Further research is warranted.

The association between cardiovascular events, cardiovascular mortality, and all-cause mortality with the baPWV has been proven in several studies, even after adjustments for the conventional risk factors, including age, gender, brachial SBP, history of use of antihypertensive agents, hemoglobin A1c, BMI, TC, HDL-C, and current smoking habits [34, 35]. The result of our analysis of the association between the baPWV and the E/A ratio without the thyroid function index was consistent with previous studies; however, the baPWV was not a significant variable after the thyroid function was included in the regression analysis. We are inclined to think that the effect of the thyroid on the pathophysiological process of LV DD was stronger in this investigation. Further studies are required.

This study had several limitations. First, it was a single-center study, and the applicability of these results to other centers needs to be confirmed. Causal relationships could not be established due to the nature of the observational study, and the influence of unknown confounding factors and potential reverse causality could not be excluded. A strictly designed prospective study may be required to verify the conclusions. Second, the blood samples were drawn between 6 AM and 8 AM after fasting for at least 10 hours, and the intra- and inter-assay coefficient variations were below 5%, but it is important to note that the influences of diurnal variation and assay variation could not be excluded completely. Another confounder that could not be neglected was the state of the menopausal stage, which was not included in the current study. Further study is needed. Third, previous literature revealed patients with SCH present LV DD at rest and systolic and DD under effort [36, 37]. Our investigation in subjects with high–normal TSH levels showed LV DD at rest, but the assessment of systolic and diastolic function under effort is required. Because the E/A ratio would be influenced by many factors and might be pseudo-normal in patients with DD, or even with severe DD, E/e’ ratio is an important indicator for evaluating the type of DD and should be employed in future in the analysis of correlation between the thyroid function, AS, and DD. Other more valuable indices including the left atrial function and volume should also be measured in future. Finally, replacement therapy was not included in the current investigation. Levothyroxine therapy appears to ameliorate AS and cardiac dysfunction, but age, the presence of CVD, and the presence of autoimmune antibodies may influence the decision to conduct replacement therapy in subjects with high–normal TSH levels and its curative effect [37, 38].

## 5. Conclusions

In conclusion, this study may signify the association between the thyroid function, increased AS, and LV DD in subjects with euthyroidism after adjustment for confounders. It would be helpful to evaluate the benefit of replacement therapy in people with high–normal TSH levels, especially in the presence of TPO-Ab in future large, multicenter, randomized trials.

## Data Availability

The datasets used and/or analysed during the current study available from the corresponding author on reasonable request.
